# Association between Circulation Indole-3-Acetic Acid Levels and Stem Cell Factor in Maintenance Hemodialysis Patients: A Cross-Sectional Study

**DOI:** 10.3390/jcm9010124

**Published:** 2020-01-02

**Authors:** Ping-Hsun Wu, Yi-Ting Lin, Pei-Yu Wu, Hei-Hwa Lee, Su-Chu Lee, Szu-Chun Hung, Szu-Chia Chen, Mei-Chuan Kuo, Yi-Wen Chiu

**Affiliations:** 1 Graduate Institute of Clinical Medicine, College of Medicines, Kaohsiung Medical University, Kaohsiung 807, Taiwan; 970392@kmuh.org.tw (P.-H.W.); yi-ting.lin@medsci.uu.se (Y.-T.L.); wpuw17@gmail.com (P.-Y.W.); scarchenone@yahoo.com.tw (S.-C.C.); 2 Faculty of Medicine, College of Medicine, Kaohsiung Medical University, Kaohsiung 807, Taiwan; chiuyiwen@kmu.edu.tw; 3 Division of Nephrology, Department of Internal Medicine, Kaohsiung Medical University Hospital, Kaohsiung 807, Taiwan; suchle5910@gmail.com; 4 Department of Medical Sciences, Uppsala University, Uppsala 75105, Sweden; 5 Department of Family Medicine, Kaohsiung Medical University Hospital, Kaohsiung 807, Taiwan; 6 Department of Laboratory Medicine, Kaohsiung Medical University Hospital, Kaohsiung 807, Taiwan; 770149@kmuh.org.tw; 7 Division of Nephrology, Taipei Tzu Chi Hospital, Buddhist Tzu Chi Medical Foundation, and School of Medicine, Tzu Chi University, Hualien 23142, Taiwan; szuchun.hung@gmail.com; 8 Department of Internal Medicine, Kaohsiung Municipal Siaogang Hospital, Kaohsiung Medical University, Kaohsiung 81267, Taiwan; 9 Faculty of Renal Care, College of Medicine, Kaohsiung Medical University, Kaohsiung 807, Taiwan

**Keywords:** uremic toxins, indole-3-acetic acid, proteomics, mitogen-activated protein kinase cascade proteins, hemodialysis

## Abstract

Protein-bound uremic toxin is a cardiovascular (CV) risk factor for patients with end-stage renal disease. Indole-3-acetic acid (IAA) was found to be associated with CV disease but the detailed pathophysiology remains unknown. Moreover, mitogen-activated protein kinase (MAPK) signaling cascades play an important role in the pathogenesis of CV disease. Thus, we explored the association between circulating IAA levels and forty MAPK cascade associated proteins in patients undergoing hemodialysis (HD). Circulating total form IAA was quantified by mass spectrometry and forty MAPK cascade associated proteins by a proximity extension assay in 331 prevalent HD patients. Accounting for multiple testing, and in multivariable-adjusted linear regression models, circulating total form IAA levels were positively associated with stem cell factor (β coefficient 0.13, 95% confidence interval 0.04 to 0.21, *p* = 0.004). A bioinformatics approach using the search tool for interactions of chemicals (STITCH) tool provided information that IAA may be involved in the regulation of cell proliferation, hematopoietic cells, and the Janus kinase (JAK)-signal transducer and activator of transcription (STAT) signaling pathway. The knowledge gained here can be generalized, thereby impacting the non-traditional CV risk factors in patients with kidney disease. Further in vitro work is necessary to validate the translation of the mechanistic pathways.

## 1. Introduction

Cardiovascular (CV) disease is the leading cause of death in patients with chronic kidney disease [1,2]. Prospective cohort studies have emphasized the importance of renal dysfunction and proteinuria on CV disease beyond the traditional risk factors (e.g., hypertension, dyslipidemia, and diabetes) [2,3,4]. Although several non-traditional CV risk factors have been proposed, protein-bound uremic toxins are an emerging issue in this field [5]. The accumulation of indoxyl sulfate (IS), which is the most well-investigated protein-bound uremic toxin, can cause vascular dysfunction in experimental animals and humans with CKD [6,7,8,9]. The potential pathological mechanism involves aggravating inflammation and oxidative stress, impaired endothelial function and its repair system [10]. However, there is a lack of studies investigating the CV impact of other protein-bound uremic toxins, such as other indole metabolites.

Indole-3-acetic acid (IAA) is an important indole metabolite metabolized from tryptophan fermentation by the gut microbiome [11,12]. It is reported to predict mortality and cardiovascular events in CKD patients [13]. Indeed, IAA increased tissue factor expression in human endothelial cells, peripheral blood mononuclear cells, vascular smooth muscle cells (VSMCs) by aryl hydrocarbon receptor (AHR) activation [14,15,16]. In addition, IAA mediated oxidative stress by increasing reactive oxygen species (ROS) production and increased the expression of inflammatory genes (interleukin-6 (IL-6), interleukin-8 (IL-8), intracellular adhesion molecule 1 (ICAM-1), and monocyte chemoattractant protein-1 (MCP-1)) in endothelial cells [13]. IAA is also positively correlated with C-reactive protein (CRP) [13], IL-6 [17], vascular cell adhesion molecule 1 (VCAM-1) [18], and MCP-1 level [17]. IAA activates the mitogen-activated protein kinase (MAPK) pathway in different cell types [13,14,16,19,20], which are likely to play an important role in the pathogenesis of CV disease, especially in cardiac hypertrophy and cardiac remodeling after myocardial infarction [21,22]. Therefore, we hypothesized that IAA may correlate with proteins that triggered MAPK cascade and further link CV disease in CKD subjects. The proteins triggering MAPK cascade are classified according to Gene Ontology (GO) biological process and selected from the Olink cardiovascular panel. Herein, the association between circulating IAA level and MAPK cascade associated proteins was investigated in a clinical setting to explore potential new CV signaling in patients undergoing hemodialysis (HD).

## 2. Experimental Section

### 2.1. Subjects

Patients aged over 30 years of age undergoing maintenance hemodialysis (HD) for at least 90 days were recruited from two hospital-based dialysis units (Kaohsiung Medical University Hospital and Kaohsiung Municipal Hsiao-Kang Hospital) from 1 August 2016 to 31 January 2017. All participants received regular HD with high-flux dialyzers three times per week at 250 to 300 mL/min blood flow rate, 500 mL/min on dialysate flow, lasting 3.5–4 h per session and received adequate HD therapy (Kt/V > 1.2). HD participants were educated and followed the renal nutrition guideline recommendation (energy intake of 30 kcal/kg ideal body weight/day and dietary protein 1.2 g/kg/day) [23,24]. All subjects gave written informed consent form and the protocol was approved by the Institutional Review Board of Kaohsiung Medical University (KMUHIRB-E(I)-20160095 and KMUHIRB-E(I)-20180139).

### 2.2. Comorbidity and Biochemical Measurements

An electronic healthcare record system was used to access baseline records. Individual information including age, sex, the primary cause of kidney failure (hypertension, diabetes, glomerulonephritis, and others), time on dialysis, dialysis access (fistula vs. graft), medical history, medication treatment, and biochemical data for all participants was recorded. Diabetes mellitus was defined by HbA1C of 6.5% or higher or taking antidiabetic medicine. Patients with a blood pressure of 140/90 mmHg or higher or taking anti-hypertensive medicine were defined as hypertensive. Before their scheduled HD session, blood samples were obtained from patients through the arteriovenous fistula or graft before HD after overnight fasting and immediately stored in −80 °C.

### 2.3. Mass Spectrometry for Indole-3-Acetic Acid Measurement

Human serum was stored at −20 °C until analysis. The detail high-performance liquid chromatography (HPLC) tandem mass spectrometry method was used as described previously [25]. In brief, standard solutions of IAA (Sigma-Aldrich, Catalog # 15148, St Louis, MO, USA) were prepared in acetonitrile (Fisher Scientific, Loughborough, UK) and were diluted to 10,000 μg/mL as stock solutions. Calibration standards were prepared by serial dilution from 0.012 to 25 μg/mL IAA for each analytical batch and stored at 4 °C during the period of study. Chromatographic separation was conducted using a Phenomenex Kinetex C8 column (250 mm × 4.6 mm × 5 μm) at room temperature with a mobile phase consisting of 5% acetonitrile with 0.1% formic acid (solvent A) and 95% acetonitrile with 0.1% formic acid (solvent B). Total IAA levels were measured by an Agilent 1200 HPLC (Agilent Technologies, Palo Alto, CA) coupled with an API 4000Q triple-quadrupole mass spectrometer (API 4000QTrap, Applied Biosystems/MDS SCIEX, Concord, Canada) and an electrospray ionization (ESI) source in a positive ion mode. To 30 μL of serum specimen in a 1.5 ml Eppendorf tube, 270 μL of acetonitrile was added for protein precipitation. After vortex mixing and centrifugation (8 min, 13,400× *g*, 4 °C), the supernatants were transferred to clean tubes, diluted 10x in solvent A, vortex-mixed for 10 s and filtered through a 0.22 μm polyvinylidene fluoride filter into the injection vial. Then, 20 μL was injected into the LC-MS/MS system to measure IAA. All controls and fortified samples were prepared in the same manner. The multiple reaction monitoring (MRM) mode was used with the characteristic fragmentation transitions. The MRM transitions, the de-clustering potential, the collision energy, and the collision cell exit potential were measured, with data acquisition and quantitative processing accomplished using the Applied Biosystems/MDS Sciex Analyst version 1.4.2 software (Foster City, CA, USA).

### 2.4. Proteomic Profiling

Target proteins that triggered MAPK cascade were selected from the cardiovascular panel (Olink Bioscience, Uppsala, Sweden). Serum samples were analyzed with the Proseek Multiplex 96 × 96 proximity extension assay. The highly specific assay simultaneously measured forty MAPK cascade associated proteins (Table S1) using two specific antibodies per protein which pairwise bind to each protein, creating a polymerase chain reaction (PCR) sequence from attached oligonucleotide strands when both antibodies are bound to the target protein surface. Each sample contained two incubations, one extension, and one detection control to determine the lower detection limit and normalize the measurements. The relative concentration of the target protein was obtained [26] and log_2_-transformed for subsequent analysis. Each protein level was normalized by plate by setting the mean to zero and standard deviation to one within each plate and storage time. Mean intra-assay and inter-assay coefficients of variation were 4% and 10%, respectively. Normalized protein expression (NPX) values were generated from quantitative PCR quantification cycle (Cq) values, where higher Cq corresponds to lower protein abundance. Cq values (log2 scale) were corrected for technical variation by an inter-plate control.

### 2.5. Statistical Analysis

For the baseline demographic data, continuous variables were presented as mean ± standard deviation (SD) or median with interquartile range (IQR), with ordinal and nominal variables as percentages. For the first analysis phase, the associations between the total concentration of IAA with the forty MAPK cascade associated proteins were investigated using linear regression models adjusting for age, sex and multiple testing by false discovery rate (FDR) < 5%. Proteins with significant associations were investigated in the next phase. *FDR* was calculated according to the original version of Benjamini and Hochberg (1995) [27], which was the likelihood of incorrect rejection of a hypothesis. In addition, the cardiovascular proteins related to protein-bound uremic toxins were ranked by ascending *p*-value, with bootstrapped confidence intervals around the ranks. In the second phase, the independent association was investigated using multivariable-adjusted models, which included covariates age, sex, hemodialysis duration, cause of end-stage renal disease (ESRD), arteriovenous shunt type, diabetes mellitus, hypertension, dyslipidemia, antiplatelet/warfarin, anti-hypertensive drugs, diabetic treatment drugs, calcium, phosphate, high sensitivity C-reactive protein (hsCRP), and total Kt/V, all assessed at baseline. Linear associations between circulating total IAA level and selected MAPK cascade associated proteins cardiovascular proteins which were statistically significant in the first phase analysis were further investigated using linear regression splines. All the statistical methods were performed using Stata (version 15, College Station, TX, USA). Results were reported as a beta coefficient (β) with a 95% confidence interval (CI), and a two-tailed *p* < 0.05 was considered statistically significant in the second phase analysis.

### 2.6. Pathway Analysis

To link the potential mechanism between IAA and SCF, a network was constructed by the Search Tool for Interactions of Chemicals (STITCH) 5.0 tool (available at http://stitch.embl.de/) [28] to identify the biological pathways. The STITCH tool conducts enrichment analysis on an open-source database containing 500,000 chemicals, 9.6 million proteins, and 1.6 billion interactions. The database is maintained by the European Molecular Biology Laboratory, the Swiss Institute of Bioinformatics, and the Center for Protein Research. The annotation study of the whole network provides another level of understanding of disease mechanism. To find direct interactions, the network analysis of IAA and the whole protein interaction to SCF were constructed. A “combined score” was computed from the four scores of protein-chemical interactions entitled “prediction”, “experimental”, “database” and “text mining” in the STITCH tool. In addition, we only considered the shortest paths (allowing no more than five interactions with the highest confidence score > 0.9 to ensure a high level of confidence for the interaction) connecting SCF and associated protein with IAA. For this purpose, the enrichment analysis of networks related to biological processes, molecular function, and the Kyoto Encyclopedia of Genes and Genomes (KEGG) pathway was also studied using the STITCH 5.0 Resource. The statistical significance was determined by the Bonferroni corrected *p*-value < 0.05.

## 3. Results

**Figure 1:**
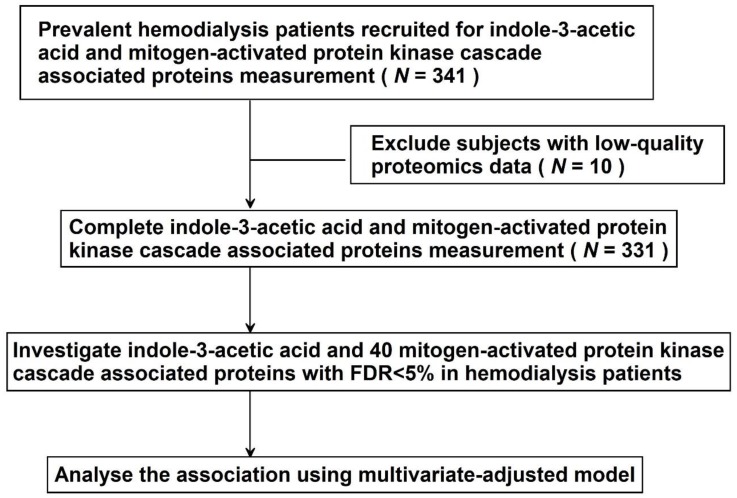
Study design.

In total, 341 HD patients were enrolled and MAPK cascade associated proteins were measured by proximity extension assays. Subjects with low-quality proteomics data (*n* = 10) were excluded. Finally, the association between total IAA level and forty MAPK cascade associated proteins was analyzed in 331 HD patients (Figure 1).

### 3.1. Demographic and Clinical Characteristics

The characteristics of the included patients are listed in Table 1. The mean age of the HD patients was 59.3 ± 11.6, 53.5% were male, 77.3% had hypertension, and 43.5% had diabetes. The major cause of ESRD was diabetes (35.3%) and glomerulonephritis (35.3%). The median (IQR) years of dialysis vintage were 5 (10) and most of the vascular access in these patients was arteriovenous fistula (87.6%). The median (IQR) serum level of ionized calcium was 4.6 (0.64) mg/dl, phosphate was 4.6 (1.4) mg/dl, hsCRP was 0.83 (2.68) mg/l and total IAA level was 0.92 (1) µg/mL (Table 1). The mean NPX value of MAPK cascade associated proteins is shown in Table S1.

### 3.2. Discovery Phase

**Figure 2:**
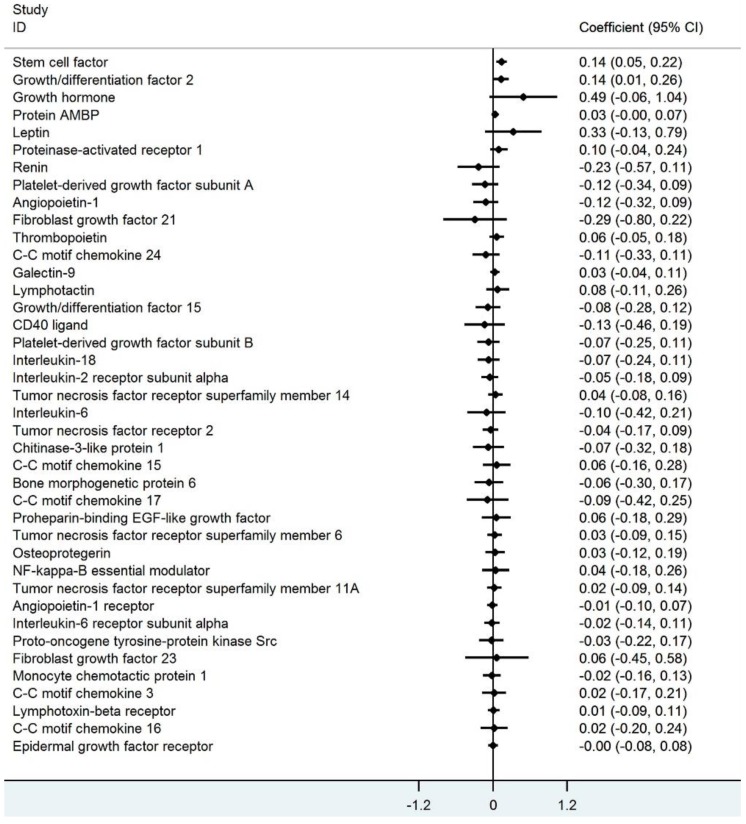
The association between Indole-3-acetic acid and 40 mitogen-activated protein kinase cascade associated proteins in linear regression models with age and sex adjustment.

**Figure 3:**
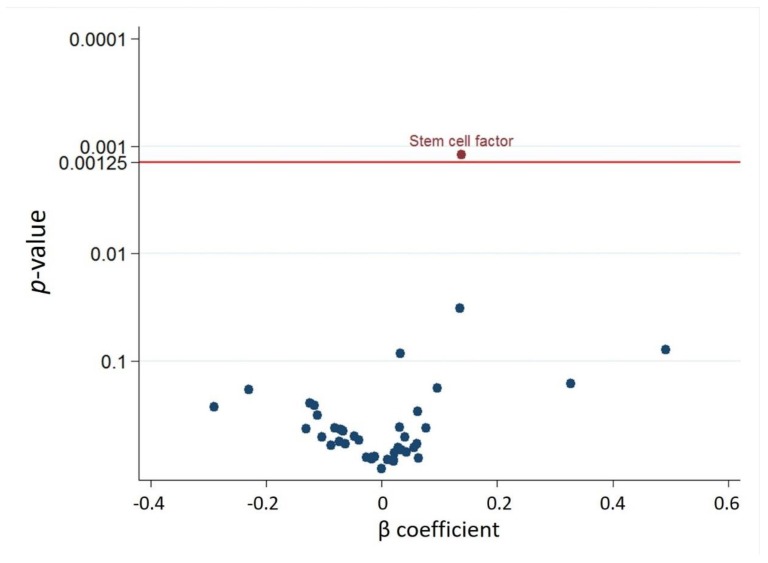
Volcano plot of the *p*-value and β coefficient for Indole-3-acetic acid and 40 MAPK cascade associated proteins association with false discovery rate <5% multiple testing control.

Regarding the association of total IAA concentration with the forty MAPK cascade associated proteins in each HD patient after adjusting for age and sex, the circulating IAA level was positively associated with stem cell factor (SCF) and growth differentiation factor 2 (Figure 2). Considering multiple testing with an FDR of 5% (corresponding to *P* = 0.00125), IAA was significantly associated with SCF (Figure 3). The ranking of the associations (with 95% bootstrap-obtained CIs) of total IAA concentration with all MAPK cascade associated proteins is graphically presented, with SCF being the top hit related to IAA, with wide confidence intervals of the ranking as expected (Figure S1).

### 3.3. Best Estimates Phase

Regarding associations between IAA and SCF in a multivariable-adjusted linear regression model, the positive association (β coefficient 0.13, 95% CI 0.04 to 0.21, *p* = 0.004) persisted after adjusting for baseline age, sex, smoking status, hemodialysis vintage, body mass index, cause of end-stage renal disease, arteriovenous shunt type, comorbidities (diabetes mellitus, hypertension, and dyslipidemia), medications (antiplatelet/warfarin drugs, anti-hypertensive drugs, and diabetic treatment drugs), and clinical laboratory data (serum albumin level, ionized calcium level, phosphate level, hsCRP level, and Kt/V) (Table 2). Linear regression spline analysis demonstrated a chiefly linear association of IAA and SCF (Figure S2).

### 3.4. A Bioinformatics Approach to Link IAA with SCF

**Figure 4:**
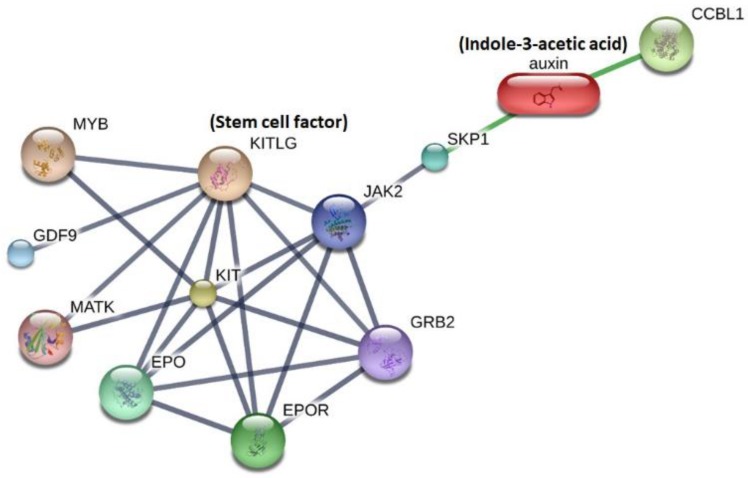
Network analysis results using the Search Tool for Interactions of Chemicals (STITCH) tool to explore the link between indole-3-acetic acid and stem cell factor.

In the literature, no study has investigated the direct effect of IAA on SCF. Therefore, a bioinformatics approach was used to explore potentially related pathways, integrating IAA with SCF in a metabolite-protein interaction network. According to the STITCH interaction network, IAA was linked to SCF (Kit ligand) through two interacting key proteins (S-phase kinase-associated protein 1 (SKP1) and Janus kinase 2 (JAK2)) (Figure 4). The functional enrichment analysis demonstrated that the link between IAA and SCF involved top biological processes (GO), including regulation of protein modification, tyrosine phosphorylation of signal transducer and activator of transcription 5 (Stat5) protein, as well as regulation of cell proliferation (Table 3). The top regulatory KEGG pathways involved included phosphoinositide-3-kinase (PI3K)/Akt signaling pathway, hematopoietic cell lineage, JAK/signal transducers and activators of transcription (JAK/STAT) signaling pathway, and cytokine-cytokine receptor interaction (Table 3). Therefore, the effect of IAA on SCF may involve PI3K/Akt and JAK/STATA pathway via two regulatory proteins (SKP1 and JAK2). These findings provide the possible mechanism to link between IAA and SCF.

## 4. Discussion

### 4.1. Principal Observations

This study investigated associations of total IAA concentration with forty circulating MAPK cascade proteins in an HD cohort. Accounting for multiple testing, and in multivariable-adjusted linear regression models, circulating total IAA levels were positively associated with SCF. A bioinformatics approach using the STITCH tool provided information that IAA may be involved in the regulation of cell proliferation, hematopoietic cells, and the JAK/STAT signaling pathway. We believe that the knowledge gained here can be generalized, thereby impacting the non-traditional CV risk factors in patients with kidney disease.

### 4.2. The Cardiovascular Damage of IAA

IAA derived from tryptophan by the gut microbiome [29] is a ligand of the AHR involved in atherogenesis, vascular inflammation, oxidative stress, and thrombogenicity [15,30]. IAA, similar to indoxyl sulfate, shares the same indole ring; however, the structure difference between IAA (a carboxyl group attached to the indole ring) and IS (a sulfate group attached to the indole ring) may lead to their specificity in the pattern of gene expression other than the classic pattern of AHR activation. In addition, levels of IS and IAA have usually been measured at the same time in clinical studies [14,31], but specific associations were demonstrated between two uremic toxins and clinical parameters, disputing a similar function in all indolic solutes [32,33,34].

IAA induces endothelial inflammation and oxidative stress *in vitro*, and is a significant predictor of mortality and cardiovascular events in a clinical study [13]. The toxic mechanism of IAA includes the production of tissue factor [14], activation of pro-inflammatory enzyme cyclooxygenase-2 [13], stimulation of inflammatory gene expression [13], inhibition of UDP-glucuronosyltransferase activity and mitochondrial activity [35], induction of endothelial progenitors cell apoptosis [32], promotion of platelet aggregation [13], and exacerbation of red blood cell procoagulant activity [36]. Interestingly, besides the potential role of AHR in atherogenesis [37], this receptor could also be involved in the imbalance between vascular damage/regeneration in CKD [38].

### 4.3. The Potential Mechanism to Link IAA and SCF

The IAA-activated nongenomic AHR/MAPK pathway [13,14,16] triggers CV disease in CKD patients. SCF, one of the important MAPK cascade proteins, known as a ligand for the receptor-type protein-tyrosine kinase KIT, plays an essential role in cell proliferation, apoptosis, differentiation and migration in several tissues. The activity of the SCF/c-KIT system is linked with the PI3K/Akt pathway, the JAK/STAT pathway, and the MAPK pathway [39]. The average serum concentration of SCF is 3.3 ng/mL in a normal human, 5–6 fold higher in CKD and HD patients [40]. SCF contributes to vasculogenesis and tissue repair by stimulating recruitment and activation of bone marrow (BM) derived, as well as tissue-resident progenitor and stem cells [41,42,43]. Interestingly, high serum indole-3-acetic acid levels were associated with low CD34^+^ and CD133^+^ endothelial progenitor cells involved in vessel repair and neovascularization [32]. In addition, AHR inhibition promotes the expansion of CD34 hematopoietic stem cells ex vivo [38]. One could hypothesize that indolic uremic solutes, by activating AHR, promote a diminution of endothelial progenitor cells, leading to a deleterious effect on the endothelial repair. SCF, which is involved in vascular repair [44,45], stimulates c-Kit to promote survival, migration, as well as capillary tube formation in endothelial cells [46] and attenuates vascular smooth muscle cell apoptosis [47]. Taken together, IAA may trigger CV risk in patients with CKD. SCF may play concurrent cytoprotective factors [48] on the CV system. In our pathway analysis, we also found a link between IAA and SCF via interacting proteins SKP1 and JAK2, but this requires further investigation to validate the translation of the mechanistic pathways suggested in vitro. Further clinical and basic research is needed to establish the interaction effect between IAA and SCF in the dialysis populations. The IAA and CV outcomes association stratified by circulating SCF levels could be a clinically relevant approach. In addition, the burden of IAA could be lower through dialytic and non-dialytic strategies, such as enhancement of solute removal, devices for extracorporeal absorption, reduction of gastrointestinal absorption, and maintenance of residual renal function [14]. Since IAA is derived from the breakdown of dietary tryptophan by gut microbiota, the production can be suppressed by restricting specific dietary protein intake, manipulating the colon microbial metabolism, or reducing intestinal absorption, in order to decrease the circulating IAA level. For example, AST-120 treatment attenuated protein-bound uremic toxin accumulation in multiple organs [49], so there is a potential treatment effect for modifying IAA associated CV toxicity.

### 4.4. Strengths and Limitations

The present study used a proximity extension assay proteomics chip to detect selected proteins in the hemodialysis population, which allowed a rapid high-throughput analysis of high sensitivity and specificity. Nonetheless, this study had several limitations. First, the cross-sectional study design could not generate causal inference. Furthermore, the weak association between IAA and SCF should be interpreted cautiously. Second, we did not measure the free concentration of IAA. Although many studies demonstrated the free concentration of protein-bound uremic toxins (indoxyl sulfate and p-cresyl sulfate) correlated with outcomes; however, the effect of indoxyl sulfate in vitro is not influenced by albumin concentration in the media [14]. Furthermore, the experiment of mimicking in vivo conditions of indoxyl sulfate and protein binding found that the presence of human albumin did not alter the effects of IS, suggesting that cellular uptake is not hampered by protein binding [50]. Furthermore, the observed concentrations of total and free indoxyl sulfate or p-cresyl sulfate are well correlated in clinical studies [51]. Additionally, most studies investigate the total concentration of IAA and evaluate the outcome association [16,17,18,52,53,54]. Third, the protein was correlated with a single serum IAA measurement at the inclusion, without information about IAA intra-individual variability, so limitations of the results can be impacted by many factors including participants’ vascular access function, diet condition, pre-analytical sampling conditions, and variation of metabolites in human. No intra-individual variability of IAA was accessed in this study. Thus, the sequential measurement of IAA might improve data quality. Fourth, the intake of tryptophan, tyrosine, and fibers from food intake was not assessed. IAA is derived from dietary protein, so circulating IAA concentrations may be influenced by dietary protein intake. Third, current research remains exploratory because there may be an alternative pathway in IAA associated with toxicity. Thus, the proposed mechanisms through which the biomarkers may be pathophysiologically related to IAA are hypothesis-generating. Finally, the circulating IAA level was not evaluated at post HD status in this study. However, studies suggest IAA is bound to albumin with a high affinity [55], estimated as an 80% protein binding ability [56]. Protein-bound solutes were difficult to remove by HD due to a combination effect that only the free fraction can be removed, such that the overall dialyzer clearance depends on the free toxin concentration and on the speed of equilibration between bound and free fractions [57]. Although IAA could be party removed during HD sessions [56], predialysis concentrations of IAA seem to depend on residual renal function rather than on dialysis adequacy, assessed by Kt/V urea [58]. In our HD cohort, all enrolled patients were presented as anuria and limited residual renal function.

## 5. Conclusions

Circulating IAA was positively associated with circulating SCF, which contributes to cell migration/proliferation and is involved in cardiac repair. This finding may provide insights into the mechanisms that lead to higher CV risk in HD patients 

## Figures and Tables

**Table 1:** Baseline characteristics of 331 hemodialysis participants.

	*N* = 331
Age (years)	59.3 ± 11.6
Male	177 (53.5%)
Smoking	36 (10.9%)
Hemodialysis duration, years	5 (10)
Body mass index, kg/m^2^	23.6 (4.8)
Cause of ESRD	
Hypertension	36 (10.9%)
Diabetes Mellitus	117 (35.3%)
Glomerulonephritis	117 (35.3%)
Others *	61 (18.4%)
Arteriovenous shunt	
Arteriovenous fistula	290 (87.6%)
Arteriovenous graft	41 (12.4%)
Comorbidities	
Diabetes mellitus	144 (43.5%)
Hypertension	256 (77.3%)
Dyslipidemia	126 (38.1%)
Medications	
Antiplatelets/Warfarin	95 (28.7%)
Anti-hypertensive drugs	156 (47.1%)
Diabetic treatment drugs	111 (33.5%)
Laboratory data	
Albumin, g/dL	3.87 ± 0.29
Ionized Calcium, mg/dL	4.6 (0.64)
Phosphate, mg/dL	4.6 (1.4)
High sensitivity C-reactive protein, mg/L	0.83 (2.68)
Total Kt/V	1.56 (0.32)
Uremic toxin (total form)	
Indole-3-acetic acid, µg/mL	0.92 (1)

Continuous data are expressed as mean ± standard deviation or median (interquartile range) and categorical data were expressed as percentages. * Other causes of end-stage renal disease include polycystic kidney disease, tumor, systemic lupus erythematosus, gout, interstitial nephritis.

**Table 2:** Associations of circulating Indole-3-acetic acid level and other clinical parameters with stem cell factor in the multivariate linear regression model.

	β Coefficient (95% CI)	*P*-Value
Indole-3-acetic acid (µg/mL)	0.13 (0.04–0.21)	0.004
Age	−0.0006 (−0.0003–0.002)	0.66
Sex	−0.06 (−0.12–0.003)	0.063
Smoking	0.01 (−0.08–0.10)	0.80
Hemodialysis duration (years)	−0.002 (−0.008–0.003)	0.51
Body mass index (kg/m^2^)	−0.001 (−0.005–0.002)	0.50
Cause of ESRD: Hypertension	−0.02 (−0.13–0.09)	0.76
Cause of ESRD: Diabetes Mellitus	0.12 (−0.008–0.25)	0.07
Cause of ESRD: Glomerulonephritis	0.007 (−0.08–0.09)	0.86
Diabetes Mellitus comorbidity	−0.15 (−0.26–-0.03)	0.01
Hypertension comorbidity	−0.03 (−0.11–0.05)	0.48
Hyperlipidemia comorbidity	0.03 (−0.04–0.09)	0.42
Antiplatelet/warfarin	−0.02 (−0.08–0.05)	0.60
Anti-hypertensive drugs	−0.05 (−0.11–0.02)	0.18
Diabetic treatment drugs	−0.02 (−0.12–0.09)	0.74
Albumin (g/dL)	0.16 (0.06–0.26)	0.002
Ionized Calcium (mg/dL)	−0.04 (−0.1–0.03)	0.24
Phosphate (mg/dL)	−0.01 (−0.04–0.02)	0.44
High sensitivity C-reactive protein (mg/L)	0.00009 (−0.007–0.008)	0.82
Total Kt/V	0.02 (−0.11–0.15)	0.74

**Table 3:** The functional enrichment analysis for investigating the link between indole-3-acetic acid and stem cell factor.

Functional Enrichments in Your Network			
Pathway ID	Pathway Description	Count in the Gene Set	Bonferroni Corrected *p*-Value
Biological Process (GO)			
GO:0031399	Regulation of protein modification process	8	0.000877
GO:0031401	Positive regulation of protein modification process	7	0.000877
GO:0038162	Erythropoietin-mediated signaling pathway	2	0.000877
GO:0042523	Positive regulation of tyrosine phosphorylation of Stat5 protein	3	0.000877
GO:0008284	Positive regulation of cell proliferation	6	0.00237
GO:0036018	Cellular response to erythropoietin	2	0.00237
GO:0050731	Positive regulation of peptidyl-tyrosine phosphorylation	4	0.00237
GO:0050776	Regulation of immune response	6	0.00237
GO:0048872	Homeostasis of number of cells	4	0.00276
GO:0045087	Innate immune response	6	0.00355
GO:0030218	Erythrocyte differentiation	3	0.00497
GO:0035234	Ectopic germ cell programmed cell death	2	0.00553
GO:0034101	Erythrocyte homeostasis	3	0.00608
GO:0043408	Regulation of MAPK cascade	5	0.00608
GO:0001932	Regulation of protein phosphorylation	6	0.0084
GO:0031325	Positive regulation of cellular metabolic process	8	0.00915
GO:0002376	Immune system process	7	0.0104
GO:0033033	Negative regulation of myeloid cell apoptotic process	2	0.0109
GO:0001934	Positive regulation of protein phosphorylation	5	0.0151
GO:0002768	Immune response-regulating cell Surface receptor signaling pathway	4	0.0151
GO:0043067	Regulation of programmed cell death	6	0.0151
GO:0045597	Positive regulation of cell differentiation	5	0.0151
GO:0048070	Regulation of developmental pigmentation	2	0.0151
GO:1902531	Regulation of intracellular signal transduction	6	0.0151
GO:0043069	Negative regulation of programmed cell death	5	0.0173
GO:0048015	Phosphatidylinositol-mediated signaling	3	0.0187
GO:0009888	Tissue development	6	0.0205
GO:0048568	Embryonic organ development	4	0.0208
GO:0008543	Fibroblast growth factor receptor signaling pathway	3	0.025
GO:0035162	Embryonic hemopoiesis	2	0.025
GO:0038095	Fc-epsilon receptor signaling pathway	3	0.0261
GO:0030097	hemopoiesis	4	0.0305
GO:0042517	Positive regulation of tyrosine phosphorylation of Stat3 protein	2	0.0323
GO:0044344	Cellular response to fibroblast growth factor stimulus	3	0.0323
GO:0046777	Protein autophosphorylation	3	0.033
GO:0046579	Positive regulation of Ras protein signal transduction	2	0.0346
GO:0007173	Epidermal growth factor receptor signaling pathway	3	0.0374
GO:0051347	Positive regulation of transferase activity	4	0.0374
GO:0002520	Immune system development	4	0.0403
GO:0048584	Positive regulation of response to stimulus	6	0.0403
GO:0038083	Peptidyl-tyrosine autophosphorylation	2	0.0419
GO:0048678	Response to axon injury	2	0.0468
GO:0061515	Myeloid cell development	2	0.0468
GO:0034097	Response to cytokine	4	0.048
GO:0071363	Cellular response to growth factor stimulus	4	0.0496
Molecular Function (GO)			
GO:0005126	Cytokine receptor binding	4	0.0187
Kyoto Encyclopedia of Genes and Genomes (KEGG) Pathways			
4151	PI3K-Akt signaling pathway	7	6.42 × 10^−8^
4640	Hematopoietic cell lineage	4	1.92 × 10^−5^
4630	Jak-STAT signaling pathway	4	0.00014
4060	Cytokine-cytokine receptor interaction	4	0.000836
4014	Ras signaling pathway	3	0.0142
5221	Acute myeloid leukemia	2	0.022
4917	Prolactin signaling pathway	2	0.027
5200	Pathways in cancer	3	0.027
4916	Melanogenesis	2	0.0445

## Supplementary Materials

The following are available online at https://www.mdpi.com/2077-0383/9/1/124/s1, Figure S1. Ranking proteins by *p*-value with bootstrapped confidence intervals around the ranks related to indole-3-acetic acid, Figure S2. The regression line between the log-transformed indole-3-acetic acid level and stem cell factor NPX units; Table S1. List of 40 mitogen-activated protein kinase cascade associated proteins measured by proximity extension assay. The mean NPX value and the standard deviation was presented in 331 hemodialysis patients.
