# Supplementary material for: Association between Circulation Indole-3-Acetic Acid Levels and Stem Cell Factor in Maintenance Hemodialysis Patients: A Cross-Sectional Study
Source: J Clin Med. 2020 Jan 2;9(1):124. doi: 10.3390/jcm9010124 (PMC7019261; doi:10.3390/jcm9010124)
Supplement: Supplementary file 1 [file jcm-09-00124-s001.zip › jcm-670622-suppl.docx]

**Table S1.** List of 40 mitogen-activated protein kinase cascade associated proteins measured by proximity extension assay. The mean NPX value and the standard deviation was presented in 331 hemodialysis patients.

| **Abbreviation** | **Protein name** | **NPX value ± Standard deviation** |
| --- | --- | --- |
| AMBP | Protein AMBP | 8.69 ± 0.111 |
| ANGPT1 | Angiopoietin-1 | 10.073 ± 0.603 |
| BMP-6 | Bone morphogenetic protein 6 | 5.943 ± 0.69 |
| CCL15 | C-C motif chemokine 15 | 10.294 ± 0.658 |
| CCL16 | C-C motif chemokine 16 | 8.616 ± 0.653 |
| CCL17 | C-C motif chemokine 17 | 10.227 ± 1.001 |
| CCL24 | C-C motif chemokine 24 | 5.595 ± 0.664 |
| CCL3 | C-C motif chemokine 3 | 9.004 ± 0.569 |
| CD40-L | CD40 ligand | 8.862 ± 0.999 |
| CHI3L1 | Chitinase-3-like protein 1 | 6.816 ± 0.783 |
| EGFR | Epidermal growth factor receptor | 3.59 ± 0.232 |
| FAS | Tumor necrosis factor receptor superfamily member 6 | 7.897 ± 0.351 |
| FGF21 | Fibroblast growth factor 21 | 10.134 ± 1.564 |
| FGF-23 | Fibroblast growth factor 23 | 7.983 ± 1.581 |
| Gal-9 | Galectin-9 | 9.46 ± 0.229 |
| GDF-15 | Growth/differentiation factor 15 | 8.983 ± 0.608 |
| GDF-2 | Growth/differentiation factor 2 | 8.922 ± 0.381 |
| GH | Growth hormone | 8.599 ± 1.733 |
| HB-EGF | Proheparin-binding EGF-like growth factor | 7.055 ± 0.705 |
| IL-18 | Interleukin-18 | 9.163 ± 0.519 |
| IL2-RA | Interleukin-2 receptor subunit alpha | 5.565 ± 0.41 |
| IL6 | Interleukin-6 | 4.833 ± 0.963 |
| IL-6RA | Interleukin-6 receptor subunit alpha | 12.727 ± 0.377 |
| LEP | Leptin | 7.731 ± 1.534 |
| LTBR | Lymphotoxin-beta receptor | 7.313 ± 0.295 |
| MCP-1 | Monocyte chemotactic protein 1 | 5.596 ± 0.436 |
| NEMO | NF-kappa-B essential modulator | 4.442 ± 0.654 |
| OPG | Osteoprotegerin | 5.39 ± 0.528 |
| PAR-1 | Proteinase-activated receptor 1 | 6.411 ± 0.413 |
| PDGF subunit A | Platelet-derived growth factor subunit A | 5.98 ± 0.628 |
| PDGF subunit B | Platelet-derived growth factor subunit B | 11.588 ± 0.529 |
| REN | Renin | 7.468 ± 1.02 |
| SCF | Stem cell factor | 10.633 ± 0.251 |
| SRC | Proto-oncogene tyrosine-protein kinase Src | 5.575 ± 0.586 |
| THPO | Thrombopoietin | 4.012 ± 0.352 |
| TIE2 | Angiopoietin-1 receptor | 8.07 ± 0.248 |
| TNF-R2 | Tumor necrosis factor receptor 2 | 9.418 ± 0.38 |
| TNFRSF11A | Tumor necrosis factor receptor superfamily member 11A | 10.558 ± 0.358 |
| TNFRSF14 | Tumor necrosis factor receptor superfamily member 14 | 9.221 ± 0.356 |
| XCL1 | Lymphotactin | 6.762 ± 0.554 |


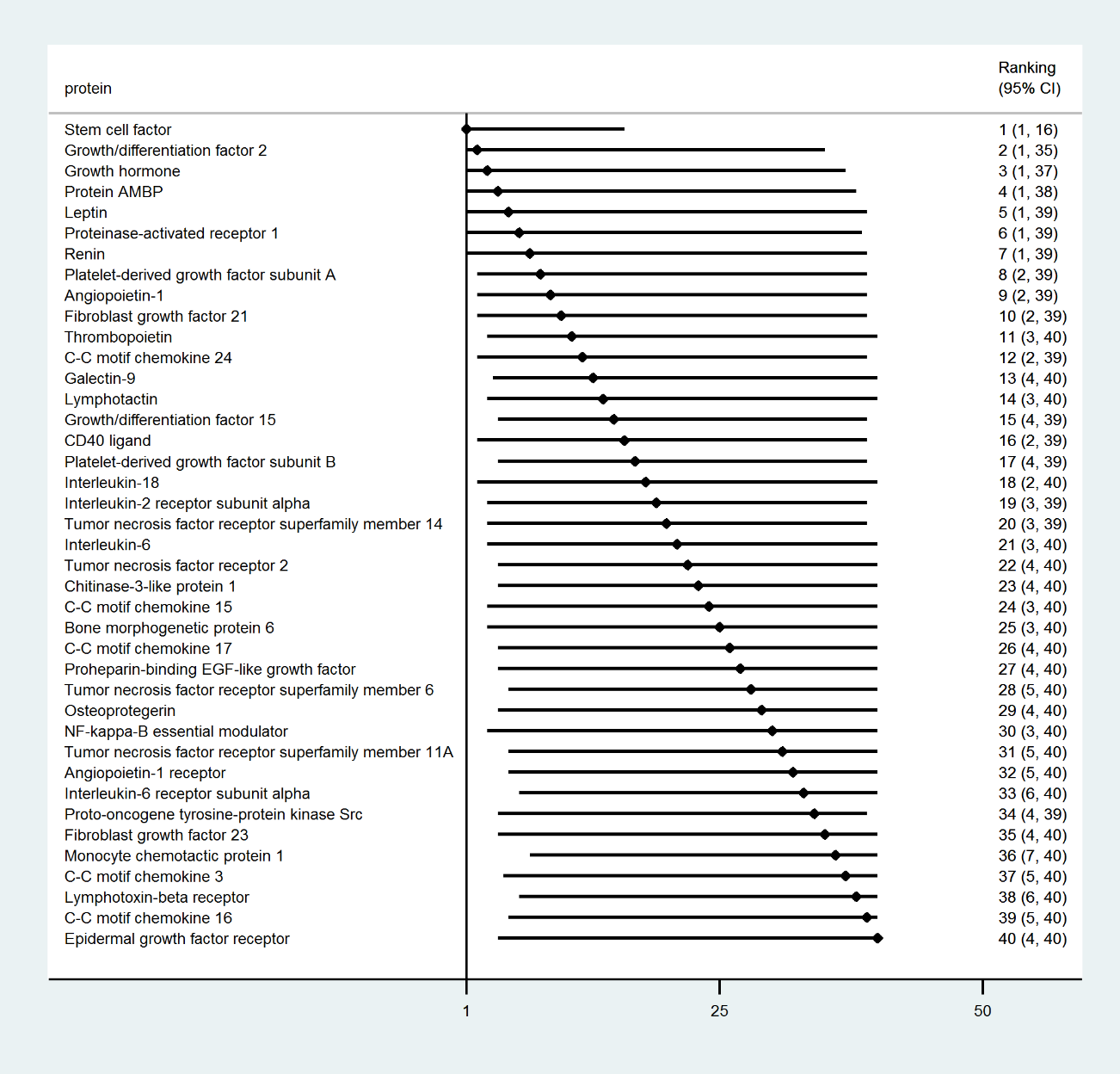


**Figure S1.** Ranking proteins by p-value with bootstrapped confidence intervals around the ranks related to indole-3-acetic acid.


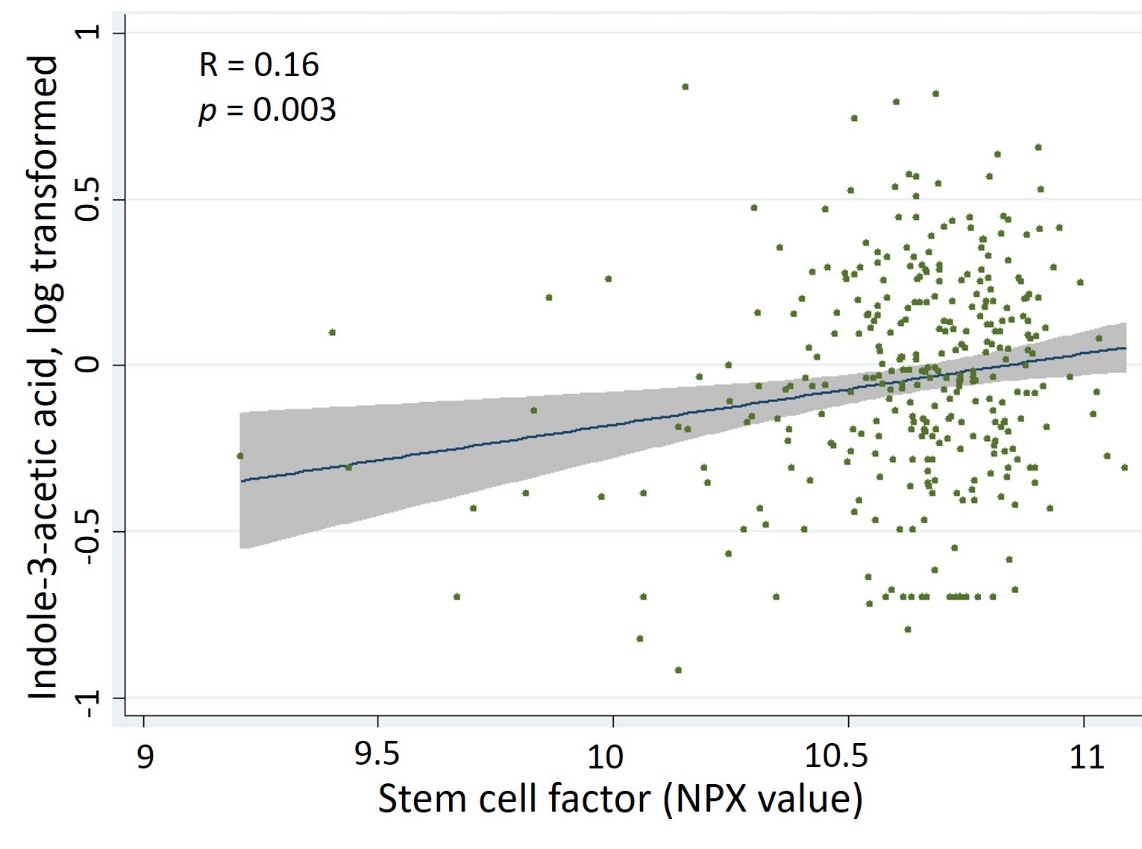


**Figure S2.** The regression line between the log-transformed indole-3-acetic acid level and stem cell factor NPX units.
